# Stewart-based characterisation of blood acid–base recovery over 48 h following exercise under hot condition in camels (*Camelus dromedarius*)

**DOI:** 10.5713/ab.250822

**Published:** 2026-03-11

**Authors:** Emad M. Samara, Khalid A. Abdoun, Mohammed A. Al-Badwi, Majdi A. Bahadi, Ahmed A. Al-Haidary

**Affiliations:** 1 Department of Animal Production, College of Food and Agriculture Sciences, King Saud University, Riyadh, Saudi Arabia

**Keywords:** Adaptation, Electrolyte Balance, Exercise Physiology, Heat Stress, Recovery Kinetics, Strong Ion Difference

## Abstract

**Objective:**

Exercise in desert heat disturbs respiratory and metabolic balance in camels. Unlike the Henderson–Hasselbalch model, which focuses mainly on bicarbonate and carbon dioxide, Stewart’s physicochemical approach clarifies acid–base regulation through strong-ion and weak-acid effects. This experiment investigated post-exercise acid–base recovery dynamics in exercise-unacclimatized dromedary camels under hot field conditions using Stewart’s approach.

**Methods:**

Five healthy bull camels completed a standardized 90-min field exercise at approximately 14 km·h^−1^ during midday heat. Recovery was monitored at baseline (2 h pre-exercise), and at 0, 3, 6, 24, and 48 h post-exercise. Sixteen respiratory, strong-ion, and weak-acid variables were measured or derived, together with biometeorological indices and ventilatory responses. Visualization tools were used to resolve temporal dynamics and inter-individual variability.

**Results:**

Heat load was greatest at PRE/0 h and again at 24–48 h, with a transiently cooler but more humid interval at 3–6 h. A triphasic recovery pattern was evident. Immediately post-exercise (0 h), respiratory rate increased sharply, while pH showed mild alkalinization with reduced partial pressure of carbon dioxide, bicarbonate, and base excess, alongside reduced oxygenation indices and early strong-ion shifts. During early recovery (3–6 h), respiratory rate declined toward baseline, but sodium excursions, hypokalemia, hypophosphatemia (p<0.05), widened apparent strong-ion difference, contracted effective strong-ion difference, and positive strong-ion gap persisted. By late recovery (24–48 h), respiratory variables largely normalized, whereas sodium instability, sustained potassium and phosphate depression (p<0.05), and renewed strong-ion gap elevation indicated incomplete systemic restoration.

**Conclusion:**

Stewart analysis revealed prolonged post-exercise disequilibrium driven mainly by persistent strong-ion and weak-acid disturbances rather than respiratory adjustment alone. In this small cohort of exercise-unacclimatized dromedary camels, recovery after exertional heat exposure cannot be assumed complete within 24 h, and environmental cooling did not ensure systemic normalization. Monitoring strong-ion and protein–phosphate domains may therefore complement conventional blood gas assessment.

## INTRODUCTION

*Camelus dromedarius* are uniquely adapted to sustain work in desert environments where high ambient temperatures and limited water availability impose severe physiological stress. Their capacity to conserve water, maintain circulation, and preserve locomotor performance makes them a valuable model for investigating acid–base regulation under arid conditions [1,2]. Nevertheless, strenuous exercise in hot environments challenges this resilience by inducing simultaneous respiratory, metabolic, and ionic disturbances. Classical studies have documented post-exercise alterations in enzymatic activity [3], biochemical and metabolic markers [4–6], thermophysiological and blood constituents [7], endocrine responses [8], and hematologic and hemorheological properties [9]. However, these reports remain fragmented, are generally restricted to observation windows of ≤24 h, and rarely integrate multiple physiological domains, leaving systemic recovery profiles in camels poorly defined.

Acid–base balance provides a central lens for understanding such recovery, as it integrates cellular metabolism, ventilatory regulation, electrolyte dynamics, and protein buffering. The conventional Henderson–Hasselbalch model, which interprets blood pH as a function of bicarbonate and carbon dioxide, has long been used in clinical and veterinary practice. However, this approach is limited in scope, as it underrepresents the roles of electrolytes, proteins, and weak acids [10]. In contrast, Stewart’s physicochemical model derives pH from three independent variables, the partial pressure of carbon dioxide (pCO_2_), the strong-ion difference (SID), and the total concentration of non-volatile weak acids (A_tot_), offering mechanistic clarity and improved diagnostic resolution [11–13]. Applications across species have shown its utility, where equine studies reveal strong-ion shifts and unmeasured anions as dominant in post-exercise recovery [14–16], and human and small-animal studies employ it to disentangle mixed respiratory–metabolic disturbances [17,18]. Recent physiological work has extended Stewart-based analyses to younger age groups of camels, revealing age-dependent modulation of acid–base balance and strong-ion variability during early postnatal development [19]. Nevertheless, no previous work has applied Stewart’s framework to adult camels or systematically examined their acid–base recovery under hot conditions.

The camel’s adaptive physiology has been proposed to support a recovery strategy distinct from that of other species. Unlike horses, which exhibit rapid post-exercise swings in lactate, bicarbonate, and pH, camels are thought in previous work to rely more heavily on controlled buffering and delayed redistribution of ions and thermal loads. The chronology of these processes, and the extent to which respiratory versus strong-ion disequilibria shape recovery in exercise-unacclimatized camels, remain unclear. Moreover, whether environmental cooling translates into systemic normalization has not been investigated.

This experiment addresses these gaps by systematically characterizing acid–base recovery over 48 h in exercise-unacclimatized dromedary camels subjected to a single standardized field-exercise protocol under hot conditions. We integrated classical blood-gas indices with Stewart-derived strong-ion and weak-acid variables and employed advanced visualization techniques, including ridgeline density plots, horizontal bar plots with curved heat lines, and deviation-from-baseline mapping, to resolve temporal patterns and inter-individual variability. This integrative approach was designed to provide a mechanistic and temporally resolved description of acid–base recovery under exertional-heat stress in exercise-unacclimatized camels. It may also generate a preliminary framework for understanding recovery in exercise-unacclimatized camels, guiding clinical monitoring and informing welfare and return-to-work protocols in similar arid environments, while recognizing that confirmation in acclimatized and differently managed animals will require further experimentation.

## MATERIALS AND METHODS

### Animals and husbandry

Five clinically healthy bull dromedary camels, aged between 4 and 6 years and weighing 350–450 kg, were enrolled. All animals were housed individually in shaded open pens (4×5 m) and provided *ad libitum* access to water and feed. Feeding consisted of alfalfa hay and Rhodes grass (*Chloris gayana* spp.), offered twice daily at 06:00 and 17:00 h, and supplemented with a commercial pelleted ration (Al-Wafi; Arabian Agricultural Services) from a single production batch. Feed was offered in amounts that ensured visual *ad libitum* availability, with refusals removed once daily; no additional electrolyte supplements, buffers, or acid–base-modifying additives were intentionally provided, and dietary cation–anion difference was not analytically determined for this cohort. Fresh water was supplied via automatic troughs and remained available *ad libitum* in the pens throughout the experiment, including during the recovery period, with access interrupted only during the exercise bout itself and brief handling for blood sampling. Veterinary examinations confirmed the animals’ health status, with no recent illness, training, or acclimation experience, and no changes in ration type or feeding schedule were introduced during the experimental period.

### Exercise protocol

To elicit a reproducible physiological load under natural hot conditions, each camel was subjected to a standardized 90-min exercise bout at approximately 14 km·h^−1^ on a sandy outdoor track during midday (12:00–14:00), coinciding with high ambient temperatures (mean 39°C) and low relative humidity (mean 15%). This workload was selected to mimic real-world exertional demands while safeguarding animal welfare [12,20]. The protocol was supervised by the same handlers to standardize effort and minimize variability, and all animals completed a single bout under identical conditions before returning to shaded pens for recovery monitoring.

### Blood sampling and laboratory analyses

Jugular venous blood samples (~5 mL) were collected in heparinized tubes and plain vacuum tubes at six standardized time points: baseline at rest (PRE, 2 h before exercise), immediately post-exercise (0 h), and at 3, 6, 24, and 48 h of recovery. Whole blood was immediately analyzed for blood gases, including pH, partial pressures of carbon dioxide (pCO_2_) and oxygen (pO_2_), bicarbonate (HCO_3_^−^), and oxygen saturation (sO_2_), using a blood gas analyzer (Rapid System, Siemens, USA). Sera were separated by centrifugation at 3,000×g for 10 min and assayed for electrolytes (Na^+^, K^+^, Cl^−^), albumin, and inorganic phosphate (PO_4_^3−^) using a semi-automatic clinical chemistry analyzer (Randox Laboratories). From these measurements, derived acid–base variables were obtained according to the Stewart physicochemical model, including the base excess (BE) (BE = 0.93×{[HCO3^−^]–24.4+14.8×[pH–7.4]}), the albumin- and phosphate-corrected anion gap (AG_norm_ = 0.20×albumin+1.50×PO_4_^3−^), the total concentration of non-volatile weak acids (A_tot_ = albumin+PO_4_^3−^), the apparent strong-ion difference (SID_a_ = Na+K–Cl–1.50), and the effective strong-ion difference (SID_e_ = [HCO3_mmolL]+A_tot), and the strong-ion gap (SIG = SID_a_–SID_e_) [13,14,21].

### Environmental monitoring and respiratory assessment

Concurrent environment conditions, ambient temperature (T_a_, °C) and relative humidity (RH, %), were logged at each time point using fixed HOBO H-08 Pro Series data loggers (Onset) positioned at camel shoulder height in the exercise and recovery areas. Respiratory rate (RR, breaths·min^−1^) was determined using a 3M Littmann stethoscope placed between the 9^th^–11^th^ intercostal spaces by counting 10 breaths and converting the recorded time to breaths per minute.

### Statistical analysis and data visualization

Data were analyzed using repeated-measures methods appropriate for a within-animal design, with Time (PRE, 0, 1, 3, 6, and 24 h) specified as the within-subject factor. Linear mixed-effects models treated Time as a fixed effect and animal as a random intercept, thereby using each camel as its own control while explicitly accounting for temporal dependence. Differences between time points were analyzed using repeated-measures ANOVA with appropriate post-hoc comparisons (Bonferroni adjustment) when applicable. A significance level of p<0.05 was adopted. Analyses were performed using SAS statistical analysis software (SAS Institute).

To visualize recovery patterns, we employed an integrated suite of graphical approaches. Ridgeline density plots were generated to illustrate distributional heterogeneity and inter-individual variability across time points. Horizontal bar plots with error bars and superimposed curved heat lines depicted the direction, magnitude, and temporal course of deviations from baseline, thereby emphasizing both effect size and persistence. Deviation-from-baseline mapping quantified the cumulative burden of deviation relative to pre-exercise values. Together, these complementary visualizations provided a multidimensional perspective of recovery, enabling simultaneous estimation of group-level responses and individual variability in exercise-unacclimatized camels subjected to exercise under hot environmental conditions.

## RESULTS

### Biometeorological indices and respiratory responses to acute exercise

Measured Ta and RH demonstrated clear temporal fluctuations across the experimental schedule (Table 1). At PRE and immediately post-exercise (0 h), Ta remained high and RH low, with both parameters statistically indistinguishable from those observed during the late recovery phase (24–48 h) (p≥0.05). A significant shift occurred during the intermediate recovery phase (3–6 h), when Ta declined while RH rose, producing the most pronounced divergence in the two indices, as confirmed by the time effect in the mixed-effects model (p<0.05). Thereafter, both gradually returned toward baseline (p≥0.05). Collectively, these dynamics indicate that the bulls were consistently exposed to hot conditions, with the greatest environmental heat load occurring at PRE/0 and again during the late recovery phase (24–48 h), while the mid-recovery interval (3–6 h) was characterized by transient relief due to cooler temperatures but higher humidity. In parallel, RR exhibited as well marked temporal fluctuations (Table 1). A sharp post-exercise tachypnea was observed (p<0.05), followed by a progressive decline during the intermediate and late phases, with values becoming statistically comparable to baseline in the model-based contrasts, indicating a near-complete recovery of ventilatory frequency within the first 24 h.

### Temporal dynamics of acid–base recovery

**Figure 1:**
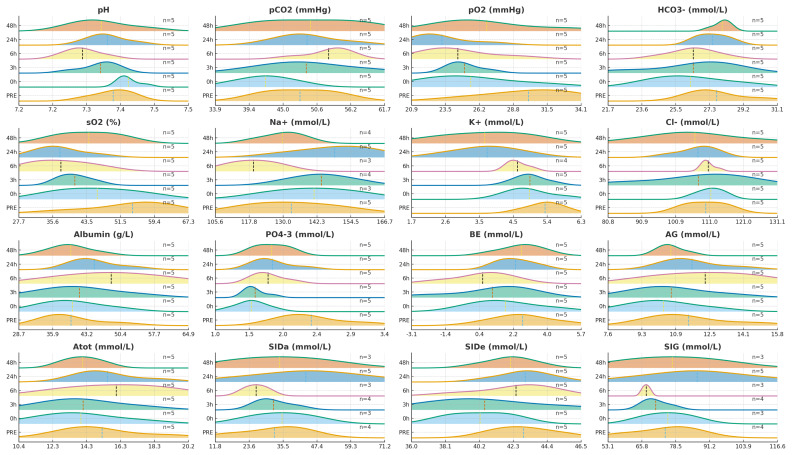
Ridgeline density plots of 16 variables central to the Stewart framework (pH, partial pressure of carbon dioxide [pCO_2_], partial pressure of oxygen [pO_2_], bicarbonate [HCO_3_^−^], oxygen saturation [sO_2_], sodium [Na^+^], potassium [K^+^], chloride [Cl^−^], serum albumin [albumin], inorganic phosphate [PO_4_^3−^], base excess of extracellular fluid [BE], anion gap [AG], total concentration of non-volatile weak acids [A_tot], apparent strong-ion difference [SIDa], effective strong-ion difference [SIDe], and strong-ion gap [SIG]) during recovery in exercise-unacclimatized dromedary camels following exercise under hot conditions. Ridgeline density plots illustrate the distribution of individual animal values across PRE, 0, 3, 6, 24, and 48 h. Dashed vertical lines denote time-specific means, and n indicates the number of animals sampled. Horizontal shifts in the density profiles reflect changes relative to baseline, whereas widening or narrowing indicates dispersion among individuals rather than statistical significance. This representation highlights both central tendency and inter-individual variability and provides a qualitative, Stewart-based visualization of acid–base recovery dynamics that complements the formal statistical analyses reported in the results.

**Figure 2:**
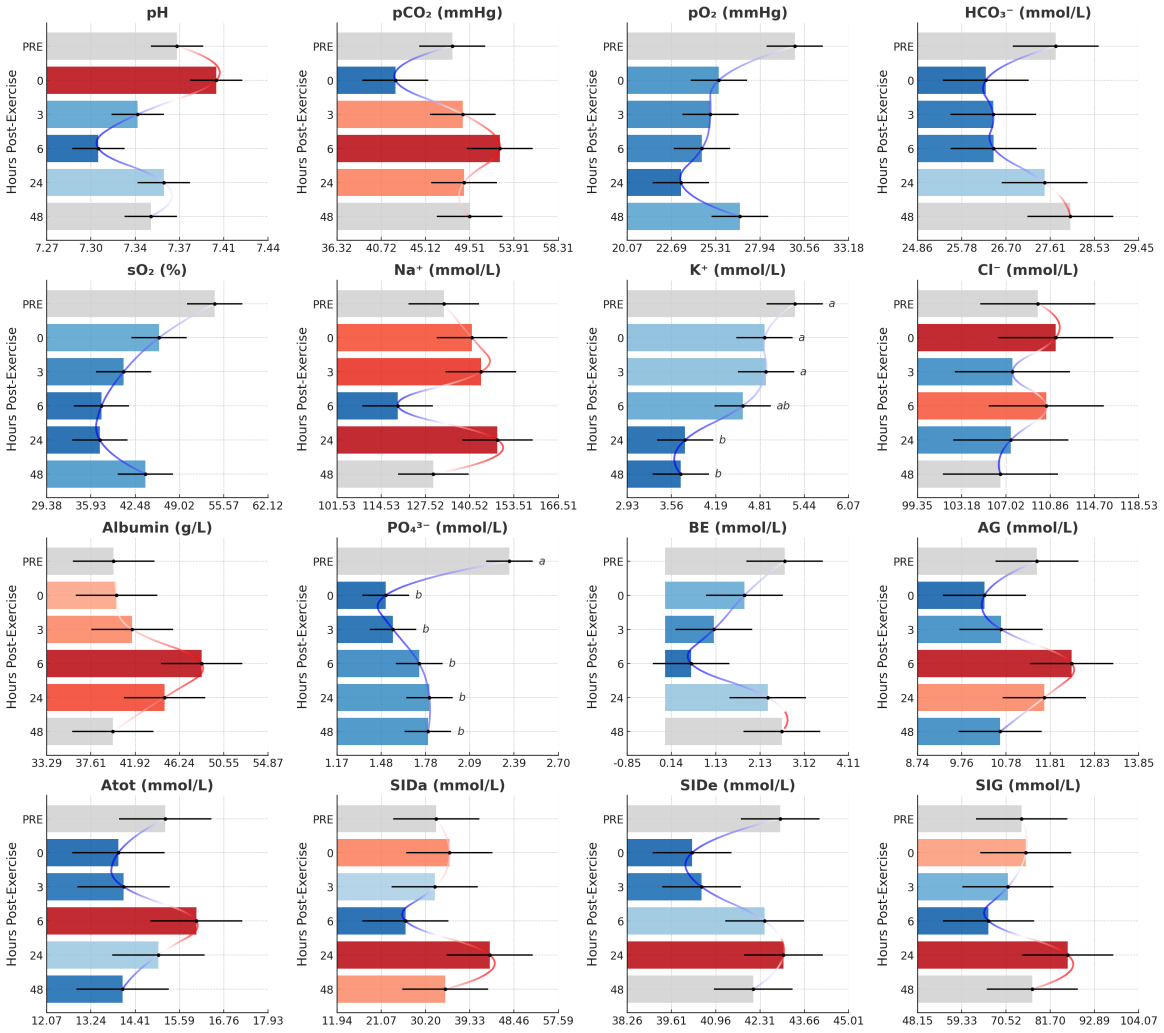
Horizontal bar plots with error bars and curved heat lines illustrating the dynamics of 16 variables central to the Stewart framework (pH, partial pressure of carbon dioxide [pCO_2_], partial pressure of oxygen [pO_2_], bicarbonate [HCO_3_^−^], oxygen saturation [sO_2_], sodium [Na^+^], potassium [K^+^], chloride [Cl^−^], serum albumin [albumin], inorganic phosphate [PO_4_^3−^], base excess of extracellular fluid [BE], anion gap [AG], total concentration of non-volatile weak acids [A_tot], apparent strong-ion difference [SIDa], effective strong-ion difference [SIDe], and strong-ion gap [SIG]) in exercise-unacclimatized dromedary camels following exercise under hot conditions. Group means±SE are shown as horizontal bars, while curved heat lines provide a visual guide to the direction and persistence of change across time points rather than representing formal regression fits. Bar length and orientation indicate the magnitude and sign of deviation from baseline (PRE), allowing comparison of recovery trajectories among variables and domains. Gray bars denote neutrality relative to baseline, blue indicates values below baseline, and red indicates values above baseline. These visual elements are intended to aid conceptual interpretation and should be considered alongside the corresponding statistical results. SE, standard error.

**Figure 3:**
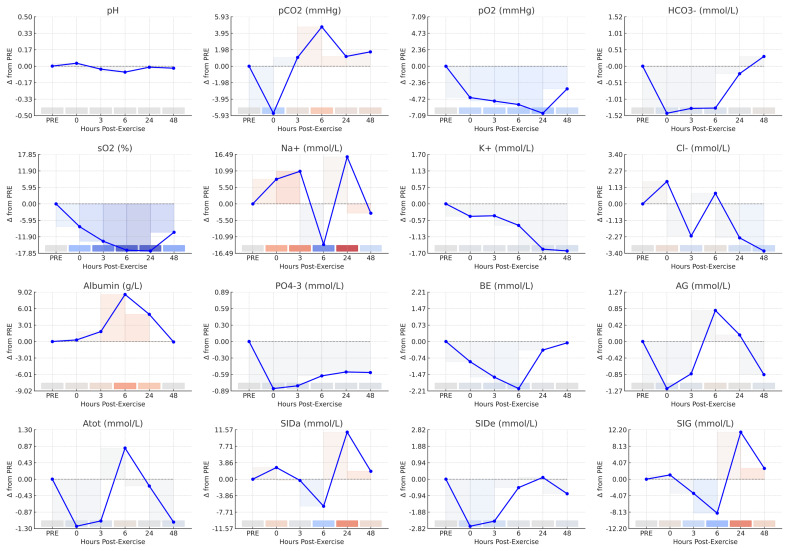
Deviation-from-baseline mapping of 16 Stewart variables (pH, partial pressure of carbon dioxide [pCO_2_], partial pressure of oxygen [pO_2_], bicarbonate [HCO_3_^−^], oxygen saturation [sO_2_], sodium [Na^+^], potassium [K^+^], chloride [Cl^−^], serum albumin [albumin], inorganic phosphate [PO_4_^3−^], base excess of extracellular fluid [BE], anion gap [AG], total concentration of non-volatile weak acids [A_tot], apparent strong-ion difference [SIDa], effective strong-ion difference [SIDe], and strong-ion gap [SIG]) during recovery in exercise-unacclimatized dromedary camels following exercise under hot conditions. Mean changes (Δ from PRE) are plotted with dotted 0-reference lines for each variable. Shaded areas represent the cumulative absolute deviation from baseline across time and serve as qualitative indicators of recovery burden rather than quantitative effect-size metrics. Larger shaded regions indicate greater persistence or magnitude of disturbance over the 48 h recovery period. Gray bars denote neutrality relative to baseline, blue indicates values below baseline, and red indicates values above baseline.

The obtained findings revealed a triphasic recovery: (i) an acute phase immediately post-exercise (0 h), (ii) an intermediate phase during early recovery (3–6 h), and (iii) a late recovery phase (24–48 h). For the main respiratory (pH, pCO_2_, BE), strong-ion (Na^+^, K^+^, Cl^−^, SIDa, SIDe, SIG), and weak-acid (phosphate, Atot) variables, the mixed-effects models identified significant time effects, and post-hoc comparisons delineated the specific phases in which values differed from baseline (Figures 1–3).

The temporal distributional patterns of the 16 measured variables, represented by ridgeline density plots (Figure 1), illustrated the course of acid–base regulation during the first 48 h of recovery in exercise-unacclimatized dromedary camels exposed to hot environment. These plots not only showed mean values but also revealed the spread and skewness of individual responses across animals and timepoints. In the acute phase, the ridgelines of pH shifted slightly upward relative to baseline (7.40 vs. 7.37), whereas pCO_2_ ridgelines contracted leftward (42.13 vs. 47.78 mmHg). HCO_3_^−^ and BE distributions narrowed but shifted downward (26.28 mmol/L and 1.78 mmol/L, respectively). pO_2_ ridgelines dropped markedly (30.00 to 25.50 mmHg), mirrored by sO_2_ reductions (54.25% to 46.01%). On the other hand, Na^+^ ridgelines moved rightward (132.95 to 141.17 mmol/L), K^+^ distributions narrowed leftward (5.31 to 4.88 mmol/L, p<0.05), while Cl^−^ showed a rightward shift (109.79 to 111.33 mmol/L). Albumin remained stable, whereas phosphate ridgelines skewed strongly downward (2.36 to 1.51 mmol/L, p<0.05). In accordance with the Stewart physicochemical framework, A_tot_, AG_norm_, SIDa, SIDe, and SIG were also traced, where A_tot_ narrowed (15.21 to 13.97 mmol/L), AG_norm_ declined slightly (11.50 to 10.29 mmol/L), SIDa ridgelines broadened rightward (32.42 to 35.15 mmol/L), SIDe distributions skewed leftward (42.93 to 40.24 mmol/L), while SIG broadened upward slightly (74.49 to 75.54 mmol/L).

During the intermediate phase, ridgelines for pH shifted downward (7.34 to 7.31), while pCO_2_ skewed strongly rightward (48.83 to 52.50 mmHg). HCO_3_^−^ remained stable (~26.4 mmol/L), but BE narrowed further, reaching its lowest point at 0.58 mmol/L at 6 h. pO_2_ ridgelines skewed further downward (24.50 mmHg), with sO_2_ showing a broad left tail (37.50%). Additionally, Na^+^ ridgelines peaked sharply rightward at 3 h (143.81 mmol/L) before collapsing leftward at 6 h (119.39 mmol/L), Cl^−^ showed slight broadening (107.58–110.52 mmol/L), while K^+^ shifted lower (4.90–4.57 mmol/L, p<0.05). Albumin ridgelines broadened markedly to the right (48.41 g/L), phosphate stayed suppressed (1.56–1.74 mmol/L), and Stewart-derived variables showed A_tot_ rising (16.03 mmol/L), AG_norm_ peaking (12.30 mmol/L), SIDa contracting sharply (26.09 mmol/L), and SIG distributions narrowing leftward (66.11 mmol/L) (Figure 1).

In the late recovery phase, ridgelines of pH shifted back toward baseline (7.36–7.35), though not fully converging, pCO_2_ remained skewed rightward (48.95–49.50 mmHg), HCO_3_^−^ broadened upward (27.50–28.03 mmol/L), and BE distributions narrowed back toward baseline (2.30–2.62 mmol/L). In addition, pO_2_ and sO_2_ ridgelines remained left-skewed (23.25–26.75 mmHg; 37.25%–44.00%). Na^+^ ridgelines showed wide variability, peaking at 148.65 mmol/L at 24 h before collapsing to 129.84 mmol/L at 48 h, K^+^ declined progressively (3.75–3.69 mmol/L, p<0.05), while Cl^−^ trended slightly leftward (107.45–106.55 mmol/L). Albumin ridgelines narrowed again (44.78 → 39.75 g/L), while phosphate showed partial recovery (1.81–1.80 mmol/L) but remained below baseline. Within the Stewart framework, A_tot_ oscillated (15.03–14.08 mmol/L), AG_norm_ narrowed toward baseline (11.67–10.65 mmol/L), SIDa peaked sharply at 24 h (43.44 mmol/L) before declining (34.28 mmol/L at 48 h), and SIG rose again (77.16 mmol/L) (Figure 1).

In Figure 2, the horizontal bar plots emphasized the direction and magnitude of deviations from baseline (±standard error [SE]), with curved heat lines mapping the temporal excursion. At the acute phase, bars for pH deviated positively (+0.02), while pCO_2_ deviated leftward from baseline (−5.65 mmHg). HCO_3_^−^ and BE showed small negative shifts (−1.45 mmol/L; −0.90 mmol/L), while oxygenation variables (pO_2_, sO_2_) showed large negative excursions. In contrast, Na^+^ and Cl^−^ bars extended rightward, elevating SIDa within the Stewart model, while SIDe shifted leftward. Albumin remained stable, phosphate dropped sharply affecting A_tot_, AG_norm_ was slightly lower, and SIG was slightly elevated. By the intermediate phase, pCO_2_ bars swung rightward (+1.05 to +4.72 mmHg), while pH bars swung downward (−0.03 to −0.06). HCO_3_^−^ was stable, BE bars contracted to −2.10 mmol/L at 6 h. Oxygenation variables showed their largest negative bars (pO_2_ −5.50 mmHg; sO_2_ −16.75%). Na^+^ extended strongly rightward at 3 h (+10.86 mmol/L) before collapsing leftward at 6 h (−13.56 mmol/L). Cl^−^ shifted slightly, K^+^ declined, albumin bars extended far to the right (+8.59 g/L at 6 h), phosphate remained negative raising A_tot_, AG_norm_ peaked (+0.80 mmol/L at 6 h), SIDa contracted (−6.33 mmol/L at 6 h), and SIG dropped below baseline. At the late recovery phase, respiratory variables partially normalized, where pH bars approached baseline (−0.01 to −0.02), BE bars narrowed close to zero, but pCO_2_ stayed positive (+1.17 to +1.72 mmHg). Oxygenation variables remained negative. Na^+^ swung strongly positive at 24 h (+15.70 mmol/L) before collapsing negative at 48 h (−3.11 mmol/L). Cl^−^ trended negative, albumin bars remained above baseline (+4.96 at 24 h), phosphate bars recovered slightly but stayed negative modulating A_tot_, AG_norm_ narrowed, SIDa peaked positive at 24 h (+11.02 mmol/L) then fell, and SIG rose again (+2.67 mmol/L at 48 h).

The deviation-from-baseline plots showed the net distance of each variable from its PRE reference at each phase (Figure 3). At the acute phase, pCO_2_ deviation was negative (−5.65 mmHg), while pH deviation was positive (+0.02). HCO_3_^−^ (−1.45 mmol/L) and BE (−0.90 mmol/L) showed modest negative deviations. Oxygenation deviations were strongly negative (pO_2_ −4.50 mmHg, sO_2_ −8.24%). Na^+^ and Cl^−^ deviated positively, elevating SIDa, SIDe reduced, phosphate deviation was strongly negative (−0.85 mmol/L) decreasing A_tot_, AG_norm_ declined slightly, and SIG was slightly positive. By the intermediate phase, pCO_2_ deviation became strongly positive (+1.05 to +4.72 mmHg), while pH showed its greatest negative deviation (−0.03 to −0.06). HCO_3_^−^ and BE remained below baseline, oxygenation deviations peaked negative, Na^+^ flipped from positive at 3 h to negative at 6 h, with corresponding SIDa swings. AG_norm_ peaked at +0.80 mmol/L at 6 h, albumin deviation was strongly positive, phosphate stayed negative contributing to higher A_tot_, and SIG deviation dropped below baseline. At the late recovery phase, pH and BE deviations narrowed near zero, but pCO_2_ stayed positive (+1.17 to +1.72 mmHg). Oxygenation deviations remained negative, Na^+^ was highly positive at 24 h but negative at 48 h, Cl^−^ negative, SIDa peaked positive at 24 h then declined, and SIDe remained modestly displaced. Albumin deviation was still positive, phosphate showed partial correction, A_tot_ fluctuated, AG_norm_ neared baseline, and SIG deviation was strongly positive at 48 h. Within Stewart’s approach, shaded areas indicated that respiratory deviations predominated at 3–6 h, while strong-ion and weak-acid components (SID, SIG, and A_tot_) persisted at 24–48 h.

## DISCUSSION

The present experiment employed the Stewart physicochemical framework [11,13] to characterize acid–base dynamics in exercise-unacclimatized dromedary camels recovering from exercise under hot conditions. By combining classical blood-gas variables with strong-ion and weak-acid indices and integrating these with distributional analyses, temporal burden plots, and deviation mapping, we showed that recovery was asynchronous and non-uniform across respiratory, strong-ion, and weak-acid domains. This heterogeneity is broadly consistent with earlier reports in equine and bovine models, where exertional stress under heat produced lingering acid–base and electrolyte disturbances, but the persistence and magnitude of deviations in the present cohort of exercise-unacclimatized camels appear more pronounced and are consistent with, but do not directly demonstrate, previously described camelid adaptations oriented toward water conservation and thermal storage [10,14–16,22–24], although the present cohort cannot by itself confirm species-wide adaptive mechanisms.

Recent developmental investigations in camel calves have further elucidated the ontogenic foundation of this regulatory phenotype. Elkhair [19] systematically examined blood and urinary acid–base parameters during the first three months of postnatal life and reported significant age-related modulation of both pH and strong-ion indices, with younger calves displaying higher SID and A_tot_ values, along with elevated Na^+^ and albumin concentrations that declined progressively with age. These ontogenic shifts have been attributed to the gradual maturation of renal electrolyte handling and protein buffering capacity, leading to enhanced acid–base stability as development proceeds. From a physiological standpoint, such early-programmed adaptations have been interpreted as indicating that the camel’s conservative buffering and ionic redistribution strategies are established during the initial stages of life. Viewed through this ontogenic lens, the asynchronous and protracted ionic recovery observed in the present adult, exercise-unacclimatized cohort may be compatible with a hypothesized developmental blueprint that prioritizes controlled buffering and delayed ionic adjustment over rapid normalization under thermal load, but this interpretation remains speculative in the absence of comparative data from acclimatized or differently conditioned animals.

Immediately post-exercise (0 h), we observed a mild alkalinization of pH rather than a fall, accompanied by decreases in pCO_2_, HCO_3_^−^, and BE. Oxygenation indices declined, and strong-ion variables shifted with sodium elevation and potassium reduction, while chloride remained near baseline. Phosphate dropped markedly, albumin was largely unchanged, and A_tot_ declined. Within the Stewart framework, SID_a_ rose, SID_e_ fell, and SIG showed a slight increase, indicating that early disturbances were dominated by respiratory and strong-ion adjustments with a concurrent depletion of weak-acid buffering capacity. During the intermediate recovery phase (3–6 h), respiratory indices began partial convergence toward baseline; however, strong-ion and weak-acid domain disequilibria became more prominent, including persistent hypokalemia, sustained phosphate depression, and instability in SIG. These findings confirm that systemic disequilibrium extended beyond the early respiratory component, with strong-ion effects dominating the intermediate window. By 24–48 h, respiratory variables had largely normalized, but sodium fluctuations, persistent phosphate depression, and elevation in SIG suggested incomplete systemic recovery. Weak-acid contributions also remained evident, with albumin changes and delayed A_tot_ convergence prolonging disequilibrium.

This sequential pattern of disturbance, with respiratory deviations dominating immediately post-exercise and strong-ion/weak-acid disequilibria prevailing later, was revealed only through Stewart’s framework, underscoring its mechanistic clarity. If evaluated solely through the Henderson–Hasselbalch approach, these disturbances might have been summarized as a mixed respiratory–metabolic disorder with partial compensation. The Stewart model instead identified the strong-ion and weak-acid domains as the principal constraints on recovery. Comparable studies in equine endurance models have shown a much faster resolution of respiratory and strong-ion disturbances [14–16], underscoring a contrast with exercise-unacclimatized camels, whose physiology may favor controlled buffering and delayed ionic redistribution over rapid normalization, a pattern that future comparative work in acclimatized animals will need to test more directly.

One of the more striking observations was the persistence of disturbances despite environmental cooling, a pattern that aligns conceptually with our recent work in small ruminants showing that improvements in ambient thermal indices do not necessarily translate into immediate systemic physiological normalization under heat stress [25]. During the intermediate phase, ambient temperature declined while humidity rose, yet respiratory indices remained displaced and strong-ion disequilibria intensified. This mismatch between external relief and internal normalization suggests that recovery in exercise-unacclimatized camels may be influenced primarily by intrinsic ventilatory and buffering inertia rather than by ambient conditions alone, echoing recent welfare-oriented strategies that emphasize monitoring internal thermal and physiological status rather than relying solely on environmental metrics [25]. RR measurements further support this interpretation: a marked post-exercise tachypnea was evident, which only partially subsided during the intermediate recovery phase and became statistically comparable to baseline by 24 h, whereas pCO_2_ remained displaced. This decoupling between breathing frequency and blood-gas normalization indicates that delayed equilibration may be linked more to alveolar gas exchange and buffering capacity than to ventilatory frequency per se, echoing findings in human exercise and critical-care studies [26,27].

Interestingly, strong-ion variables (Na^+^, Cl^−^, SID_a_, SID_e_, and SIG) were the most labile, with hypernatremia at 24 h followed by relative hyponatremia at 48 h, reflecting unstable sodium regulation that may involve delayed renal excretion or redistribution. Horses typically exhibit transient SID perturbations that resolve quickly, and similar normalization is reported in cattle over shorter intervals [16,23]. The more protracted disturbances observed in the present exercise-unacclimatized camels are consistent with possible renal or extrarenal constraints under combined exertional and thermal stress. Weak-acid components (albumin, PO_4_^3−^, and A_tot_) added further complexity: hemoconcentration and hyperalbuminemia, together with delayed A_tot_ convergence, indicate ongoing weak-acid buffering beyond the resolution of respiratory deviations. The persistent hypophosphatemia observed across all recovery phases is particularly notable, given the central role of phosphate in intracellular buffering and energy metabolism, and may reflects prolonged sequestration within skeletal muscle, in line with earlier camel studies under exertional-heat stress [28,29]. Coupled with hypokalemia, potentially driven by adrenergic activation and aldosterone-mediated redistribution [30], these patterns suggest that electrolyte and energy-buffering systems remain perturbed long after conventional respiratory variables have largely normalized. Elevated SIG during late recovery indicates the presence of unmeasured anions, possibly organic acid intermediates of muscle metabolism, consistent with incomplete clearance of metabolic by-products.

Integrated indices such as BE and AG_norm_ further illustrate the interplay among domains. BE showed biphasic excursions, narrowing toward baseline only late in recovery, while AG_norm_ expanded early and contracted thereafter. Stewart’s approach clarified how strong-ion and weak-acid contributions collectively prolonged disequilibrium, an insight paralleling human critical-care literature where Stewart analysis exposes disturbances that conventional metrics may mask [12,13,18,31]. Importantly, Stewart’s and Henderson’s frameworks should not be viewed as mutually exclusive; their integration offers the most comprehensive picture of camel physiology, as it does in equine and human contexts. The strengths of this experiment include its integration of classical and Stewart-derived variables, the use of multidimensional visualization, and the systematic mapping of recovery over 48 h, which together enabled detection of asynchronous and domain-specific responses that would likely have been overlooked using a narrower analytic lens.

Nonetheless, several limitations warrant explicit recognition. First, the relatively small sample size (n = 5 bulls) means that the present findings should be interpreted as cohort-level patterns rather than definitive species-wide estimates. The repeated-measures design strengthens internal comparisons across time within animals, but it does not substitute for larger, more heterogeneous cohorts. Second, all animals were exercise-unacclimatized and completed a single standardized workload; therefore, the temporal profile described here reflects responses in exercise-unacclimatized camels under one specific exertional-heat scenario and should not be generalized to acclimatized animals, to different exercise intensities, or to other management systems. Third, nutritional and hydration management were standardized at the level of ration type, feeding schedule, and *ad libitum* water provision in shaded pens, but we did not perform formal chemical analysis of the feeds or calculate dietary cation–anion difference, nor did we quantify individual feed and water intake. As a result, subtle variation in DCAD or voluntary intake may have contributed to inter-individual differences in strong-ion balance and recovery profiles, and such influences cannot be excluded. Future work with larger cohorts, inclusion of both acclimatized and unacclimatized groups, graded exercise protocols, repeated exercise bouts, and quantified diet and intake will be required to test whether the triphasic recovery pattern and the dominance of strong-ion and weak-acid disequilibria observed here are consistent features of camel physiology or specific to the present experimental context.

## CONCLUSION

In conclusion, using the Stewart physicochemical framework, this experiment provides an initial, mechanistically detailed description of post-exercise acid–base recovery dynamics in a small cohort of exercise-unacclimatized dromedary camels exposed to a single standardized field-exercise protocol under hot desert conditions. Recovery was triphasic and asynchronous, with early disturbances dominated by respiratory and oxygenation changes, followed by a prolonged period in which strong-ion and weak-acid disequilibria persisted despite environmental cooling and partial normalization of blood-gas variables. The sustained hypophosphatemia, hypokalemia, sodium instability, and elevated SIG indicate that electrolyte and weak-acid domains remain perturbed long after conventional respiratory indices appear near baseline, suggesting that intrinsic buffering and ionic redistribution mechanisms may represent important constraints on recovery in this context. Clinically, the data suggest that, for exercise-unacclimatized camels exposed to comparable exertional-heat loads, full systemic recovery cannot be assumed within 24 h and that monitoring should extend beyond pH and pCO_2_ to include strong-ion and phosphate-related variables. While the small sample size and absence of an acclimatized comparison group limit broader generalization, the present findings offer a hypothesis-generating framework for future comparative and interventional work aimed at improving camel welfare, veterinary care, and work-readiness in hot environments.

## Figures and Tables

**Table 1:** Temporal changes in ambient temperature, relative humidity, and respiratory rate of dromedary camels before and after acute exercise under hot conditions

Time	Parameters		

	Ambient temperature (°C)	Relative humidity (%)	Respiratory rate (breath/min)
2 h-PRE	39.11±2.13^a^	15.24±0.14^c^	16.18±0.22^d^
0 h-POST	39.16±0.55^a^	15.10±0.11^c^	50.36±2.01^a^
3 h-POST	36.02±1.38^b^	21.74±0.18^b^	25.05±2.79^b^
6 h-POST	29.41±0.89^c^	31.40±0.15^a^	20.15±1.10^c^
24 h-POST	39.13±0.89^a^	15.12±0.13^c^	20.38±3.63^c^
48 h-POST	39.21±1.03^a^	14.84±0.12^c^	17.66±0.26^cd^
p-value^1)^	<0.001	<0.001	<0.001

Values are presented as mean±standard deviation.

1) p-values represent the overall effect of time derived from linear mixed-effects models.

a–d Different superscript letters within a column indicate significant differences between time points (p<0.05).

## Data Availability

Upon reasonable request, the datasets of this study can be available from the corresponding author.
